# Assessing the Effects of Dasatinib on Mesenchymal Stem/Stromal Cells

**DOI:** 10.1007/s12195-024-00830-1

**Published:** 2024-10-29

**Authors:** David P. Heinrichs, Vitali V. Maldonado, I. Kade K. Ardana, Ryan M. Porter, Rebekah M. Samsonraj

**Affiliations:** 1 Department of Biomedical Engineering, Engineering Research Center, College of Engineering, University of Arkansas, 700 W Research Center Boulevard, Fayetteville, AR 72701 USA; 2 Cell and Molecular Biology Interdisciplinary Program, University of Arkansas, Fayetteville, AR 72701 USA; 3 Department of Orthopedic Surgery, University of Arkansas for Medical Sciences, Little Rock, AR 72205 USA

**Keywords:** Mesenchymal stem cells, Senescence, Senolytics, Dasatinib, Cell therapy

## Abstract

**Introduction:**

Progressive aging, or senescence, of mesenchymal stem/stromal cells (MSCs) is a major obstacle faced when trying to culture potent stem cells for use in therapy. Senescent cells are irreversibly nondividing cells that cease performing critical functional effects. Elimination of senescent cells using biochemical means, such as the use of senolytic drugs like dasatinib, may be useful in retaining the viable and proliferating populations of the cells.

**Methods:**

An in vitro approach was used to investigate the effect of dasatinib on phenotypic, genotypic, and immunomodulatory functionality of osteogenic and adipogenic differentiated MSCs. Replicative senescence was achieved through multiple sub-culturing in vitro, then senescent and non-senescent cultures were treated with a standard dosage of dasatinib. MSCs were then differentiated into osteogenic, adipogenic or chondrogenic cultures using conditioned media to be tested for the three criteria being investigated.

**Results:**

Significant changes were observed in these criteria, indicated by evidence gathered from proliferation and indoleamine 2,3 dioxygenase activity assays. Phenotypic results of dasatinib were shown to reduce the population of senescent MSCs while allowing non-senescent MSCs to continue differentiating and proliferating without interference from senescent cells. Genotypic results showed no change to upregulation in markers associated with osteogenic and adipogenic cells when exposed to dasatinib. Indoleamine Dioxygenase activity showed insignificant differences in cells exposed to dasatinib versus control groups, providing evidence against compromised cellular immune function.

**Conclusion:**

This investigation provides insight into how dasatinib effects MSCs functional ability and provides a better understanding of the function of senolytic agents.

## Introduction

Mesenchymal stem/stromal cells (MSCs) are multipotent stem cells that are characterized by their ability to adhere to plastic, express cell surface markers CD105, CD73 and CD90, and lack surface markers CD45, CD34, CD14 or CD11b, as well as the ability to differentiate into osteoblasts, adipocytes, and chondroblasts [1]. High-growth capacity MSCs also exhibit the lack of the gene encoding glutathione *S*-transferase theta 1 (GSTT1), making GSTT1 a genomic DNA biomarker for MSC scalability [2]. In addition, MSCs have been shown to secrete growth factors and other soluble molecules such as interleukins, cytokines, and chemokines that play critical roles in tissue repair and regeneration. Owing to their self-renewal, regenerative and immunomodulation properties, MSCs function as medicinal signaling cells by virtue of their secretion of soluble factors and proteins that are anti-inflammatory, anti-fibrotic, anti-microbial, anti-apoptotic, and pro-regenerative [3].

Of the several tissue sources of human MSCs, bone marrow derived MSCs are particularly advantageous owing to their widespread use in research as well as their ability to differentiate into cells of the mesoderm lineage [4]. In cellular therapy applications, bone marrow derived MSCs have been used in more clinical trials than any other MSC source [5]. For these reasons, we used bone marrow derived human MSCs for all following experiments.

The *in vitro* ability of MSCs to successfully differentiate into osteogenic, adipogenic, and chondrogenic lineages is a measure of their phenotypic characteristics. Potency is defined as the ability of MSCs to exert functional effects *in vivo* or within an *in vitro* functional model. Our study specifically adheres to the criteria established by the International Society for Cellular Therapy (ISCT) as well as additional criteria from Samsonraj et al. to define potency [1, 6]. In this study, we assessed potency using an *in vitro* functional model to test critical features of MSCs including differentiation and immunosuppression.

A major obstacle in the use of MSCs for cellular therapy is replicative senescence, or progressive aging. In 1961, Hayflick and Moorhead first described cellular senescence as an irreversible nondividing state where cells remain metabolically active after reaching their replicative potential *in vitro* [7]. Cellular senescence can be triggered in response to a variety of stressors, including telomere shortening, oxidative stress, DNA damage, and oncogene activation leading to an overall decline in potency [8, 9]. In MSC research, cells are extensively sub-cultured or expanded in the laboratory which results in some cells within the population undergoing senescence.

Senolytics are drugs that specifically target senescent cells by inducing apoptosis of senescent populations while not affecting non-senescent cells. Our objective in this study was to test how dasatinib, a known senolytic, affects the potency and viability of senescent MSCs. Dasatinib is an FDA approved drug used for treatment of chronic myeloid leukemia [10] and has been suggested for treatment of gastric cancer [11]. Furthermore, dasatinib has been identified as being potentially senolytic [12, 13] based upon its predicted ability to transiently disable the senescent cell anti-apoptotic pathways network [14], and has been shown to promote senescence clearance in irradiated mouse fibroblasts [15]. More specifically, dasatinib is a Src/tyrosine kinase inhibitor that interferes with members of the ephrin survival-regulating dependence receptors (ENFB), and appears to exhibit specificity in terms of clearing senescent preadipocytes [16, 17]. Elimination of senescent cells (in MSC expansion cultures) using biochemical means, such as the use of senolytic drugs, may be useful in retaining the viable and proliferating populations of the cells, and together improve overall MSC functionality.

## Materials and Methods

### Culture of MSCs

Commercially sourced (RoosterBio) bone marrow-derived MSCs were cultured in complete maintenance media (Dulbecco's modified Eagle's medium [DMEM], 1 g/l glucose, 10% fetal calf serum [FCS], 2 mM l‐glutamine, 50 U/ml penicillin, and 50 U/ml streptomycin) with a media change every 3–4 days, and routinely passaged upon 85% confluence using 0.125% trypsin. Cells from multiple donors were cultured and maintained in a humidified incubator at 37 °C with 5% CO_2_. Senescent MSCs were generated by repeated subculturing. Two different age groups of MSCs were cultured, passages 5–9 for younger, non-senescent, cells and passages 15–20 for older, senescent, cells. Each donor’s cultured MSCs were split into four experimental groups as follows: non-senescent cells treated with dasatinib, non-senescent cells normalized with Dimethyl Sulfoxide (DMSO), senescent cells treated with dasatinib, and senescent cells normalized with DMSO. All experimental groups were performed in triplicate for technical replicates.

### Live/Dead Staining with Nuclear Counterstain

Live/dead staining was performed following ThermoFisher Scientific LIVE/DEAD viability/cytotoxicity assay kit (L32250). Multiple senescent cell cultures were treated with varying concentrations of dasatinib or DMSO from 0.2 to 20 µM for 72 h. This timepoint is the minimum amount of time needed for dasatinib to have a significant effect on cell populations in culture, determined experimentally. The cultures were then washed with PBS and the conditioned media was replaced with red and green dyes diluted in a working reagent. The cells were incubated for 30 min at room temperature with DAPI solution being added in the last 15 min to stain cell nuclei. Cells were washed again and imaged using a fluorescent microscope. ImageJ was used to analyze fluorescent cells.

### Growth Rate Analysis

MSCs were seeded into 12 well plates at 5000 cells per cm^2^ in maintenance medium until ~ 80% confluence. The cells were then treated with 2 µM of dasatinib or DMSO for 72 h. Each day after the end of treatment, cells from a well of the plate were detached and transferred to a well of a 96-well plate. The cells were introduced to a Cell counting Kit-8 assay (Tocris Cat. 7368) for 3 h, then absorbance readings were taken at 450 nm on a plate reader. This process was repeated for a total of 6 days post-treatment. Simple linear regression was performed on each experimental group to determine the overall rate of growth.

### Senescence β-Galactosidase Staining

Senescence β-galactosidase (SABgal) staining was performed following a kit protocol from Cell Signaling Technology (Catalog #9860). MSCs were seeded into 12-well plates in maintenance media and allowed to grow to ~ 80% confluence. An experimental group of wells was exposed to 2 µM of dasatinib in solution with DMSO while a control group was exposed only to DMSO for 72 h. After the exposure time, the media was aspirated and replaced with maintenance media to allow cells to recover for 96 h. At the endpoint of the recovery, cells were washed with 1 × PBS, and treated with the SABgal kit according to manufacturer’s recommendations. The wells were imaged at 100× magnification for β-galactosidase activity. Cell images were manually counted for senescent and nonsenescent populations using ImageJ. Two-tailed *t*-tests were performed between treated and control cells to find statistical relevance within each passage.

### Osteogenic-Specific Collagen Plate Preparation

Osteogenic plate preparation was conducted using Advanced BioMatrix Type I bovine collagen solution (Cat. 5005) diluted to 50 µg/ml. Every plate used for osteogenic differentiation was coated with the collagen solution for 3 h then aspirated, washed with PBS, and left to dry under a sterile fume hood for 12 h before being seeded with MSCs.

### Osteogenic Differentiation

Osteogenic differentiation was performed by seeding the 4 experimental groups in 6-well plates at 3000 cells per cm^2^ for the lower passages, and 5000 cells per cm^2^ for higher cell passages in maintenance medium. The cells were allowed to attach and grow in the 6-well plates for 3–4 days before changing to osteogenic media (maintenance media, 10 nM dexamethasone, 25 μg/ml ascorbic acid, and 10 mM β‐glycerophosphate). The experimental wells of each plate had dasatinib in solution with DMSO mixed into the osteogenic medium at 2 µM per mL and the control wells were mixed with only DMSO at 2 µM concentration. Osteogenic media with DMSO or dasatinib treatment was replaced every 3–4 days for 7 days. On day 7, the dasatinib treatment and DMSO control were no longer added to the osteogenic media and the regular osteogenic media replacement was continued for another 14 days to allow the cells to continue differentiating. After 21 days total, the cells were fixed to the plate with a 4% formaldehyde solution then stained with Alizarin Red to visualize differentiation and mineralization on the plates.

### Osteogenic Quantification

Osteogenic plates were analyzed for stain concentration and DNA content after imaging. Each well was introduced to 10% acetic acid for 30 min to detach all contents from the plate and transferred to microcentrifuge tubes. Cells were vortexed, heated to 85 °C for 10 min, and then transferred to ice to cool. The tubes were centrifuged at 20,000×*g* for 15 min and the supernatant was collected for visualization. 10% ammonium hydroxide was added to the supernatants to neutralize the pH and absorbance reading were read at 405 nm on a plate reader. The leftover cell pellet was lysed, and DNA was isolated using the Qiagen DNA mini kit (Cat. 51306). The DNA was quantified using a nanophotometer. Two-tailed *t*-tests were performed between treated and control cells to find any statistically significant difference between the control and treatment groups of each passage.

### Adipogenic Differentiation

Adipogenic differentiation was performed by seeding the four experimental groups in 6-well plates at 3000 cells per cm^2^ for the lower passages, and 5000 cells per cm^2^ for higher cell passages in maintenance medium. The cells were allowed to attach and grow in the 6-well plates for 3–4 days before changing to adipogenic media (high glucose DMEM supplemented with 10% FBS with 1 µM dexamethasone, 10 µM insulin (SAFC-91077C), 100 µM indomethacin (Sigma I7378), 11.5 µg/ml 3-isobutyl-1-methylxanthine (Sigma-Aldrich I5879). As with osteogenic differentiation, the experimental wells of each plate had dasatinib in solution with DMSO mixed into the adipogenic medium at 2 µM and the control wells were mixed with only DMSO at 2 µM concentration. Adipogenic media with dasatinib or DMSO was replaced every 3–4 days for 7 days. On day 7, the dasatinib treatment and DMSO were no longer added to the adipogenic media, and the regular adipogenic media replacement was continued for another 21 days to allow the cells to continue differentiating. After 28 days total, the cells were fixed in 4% formaldehyde then stained with Oil Red O to visualize the lipid globules on the plates. Stained images were quantified using ImageJ.

### Gene Expression Analysis

Relative quantification of real-time reverse transcription combined with polymerase chain reaction (RT-qPCR) [18] was performed on RNA components of lysed osteogenic and adipogenic cells. MSCs were cultured and seeded in 6-well plates and treated with osteogenic media or adipogenic media the same way as stated in previous sections. The plates were exposed to 2 µM concentration of dasatinib or 2 µM of DMSO for 7 days then allowed to continue differentiating in normal adipogenic or osteogenic media for another 7 days with media replacement every 3–4 days. On day 14, the media was aspirated from the wells and the cells were lysed with RLT lysis buffer. The cell lysate was collected, and RNA was isolated using the Qiagen RNeasy kit (Cat. 74104). Collected RNA was diluted to normalize the concentration per microliter, then converted into cDNA on a thermocycler using VILO Superscript cDNA synthesis kit (Invitrogen Ref. 11754-250). Primers were added to the cDNA, then qPCR was performed using a thermocycler with SYBR green as a probe. Osteogenic markers tested were ALP, COL1A1, SP7, and IBSP. Adipogenic markers tested were FABP4, CEBPα, and PPARγ. Relative quantification analysis was done using the comparative CT method. Relative expression units (REU), or ddCt values, were measured with RPLP1 or GAPDH as a housekeeping gene. Multiple* t*-tests were used to find any statistically significant difference between the control and treatment groups of each passage.

### Chondrogenic Differentiation

MSCs were seeded into a round-bottom 96-well plate at a density of 250,000 cells per well in maintenance media. The plate was centrifuged (200 rcf for 10 min) to induce pellet formation and left to incubate overnight. After 24 h, maintenance media was aspirated and 200 µL of chondrogenic differentiation media (Glibco Ref. A10069-01) was added to each well. MSCs from senescent and nonsenecent groups were split into three subgroups for differentiation: treatment without dasatinib, treatment without dasatinib and with transforming growth factor beta (TGF-β) (10 ng/mL), and treatment with dasatinib and TGF-β (10 ng/mL). Each group was treated for 7 days with media changes every 3–4 days, then all groups were exposed to normal chondrogenic differentiation media for another 21 days. After a total of 28 days, the cell pellets were fixed in 4% formaldehyde, stained with toluidine blue, sectioned, and imaged.

### Indoleamine 2,3-di-oxygenase (IDO) Activity Assay

The IDO assay was performed using an IDO Elisa kit (MyBioSource MBS028821). MSCs were seeded into 12-well plates at 3000 cells per cm^2^ for the lower passages and 5000 cells per cm^2^ for higher passages in maintenance medium and the cells were grown to 75–80% confluency. The same four experimental groups were used with dasatinib and DMSO treatments, however we also treated the wells with 50 ng/ml interferon gamma (IFN-γ), a cytokine that is known to play a role in activating immune response, or a 10% Fetal Bovine Serum (FBS) in PBS solution. The FBS solution is what IFN-γ is dissolved in and acts as a control for the treatment. After 72 h, the conditioned media of the treatment was collected for IDO assay and the cells were lysed using RIPA buffer to obtain a cell lysate that was also collected. Data from both cell lysate and supernatant was gathered, then combined to find the total concentration of each treatment group. A one-way ANOVA was performed across all data in each donor to find any significant differences throughout all 8 groups.

## Results

### Dasatinib Selectively Clears Senescent Cells

**Fig. 1:**
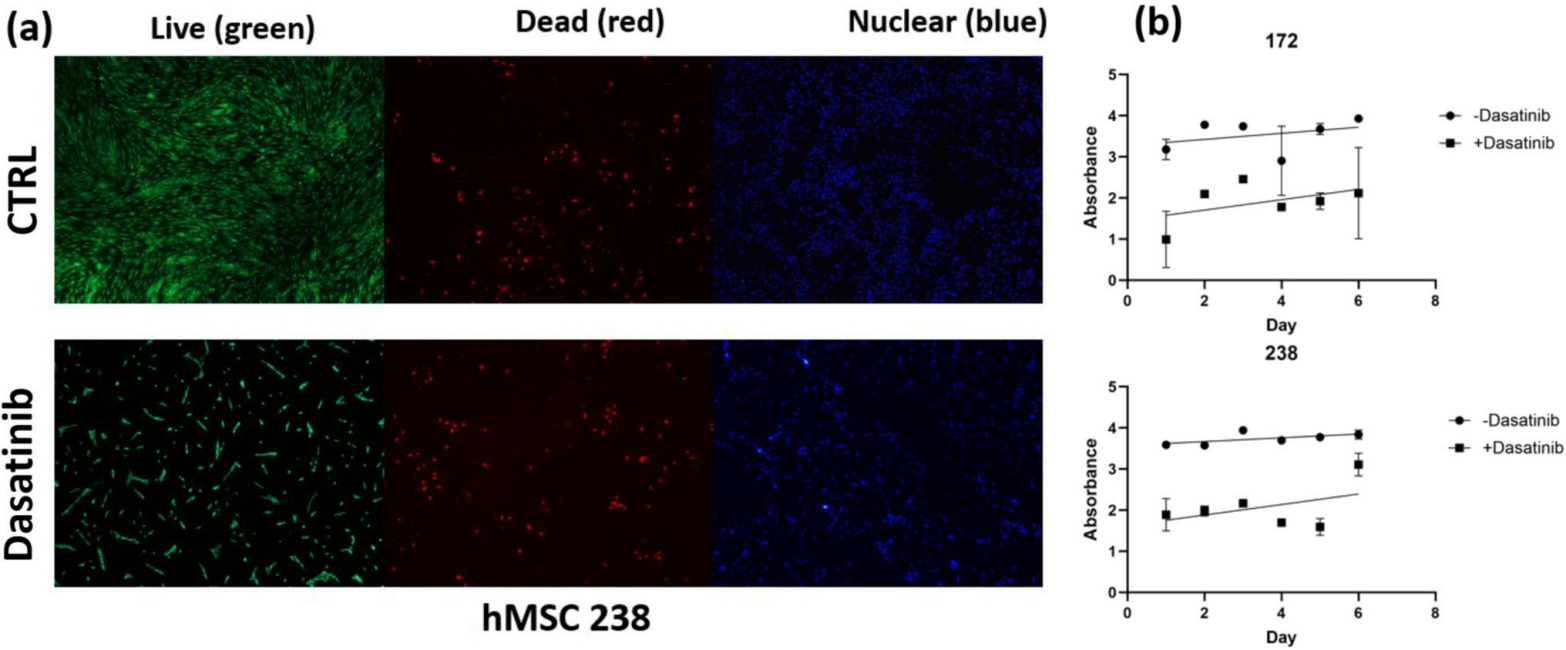
**a** Live/dead stain images of MSCs exposed to 2 µM of dasatinib. Any living cells are fluorescent green on the left images, any dead cells show as red in the middle, and cell nuclei are shown as blue on the right. **b** Growth curve analysis of the 6 days following treatment of cells exposed to 2 µM of dasatinib

The live/dead staining results (Fig. 1a) showed a decrease in live cell number when exposed to dasatinib as opposed to the negative control group for all concentrations of dasatinib. Qualitative image analysis shows there are 998 live cells per field of view in the control group while after treatment the cell count decreases to 297 per field of view. This equates to a 70.2% decrease in live cell number post treatment with no recovery time. The numbers of dead cells are 240 and 192 for the control and treatment groups respectively, equating to a 20.0% decrease in dead cell numbers after treatment.

**Fig. 2:**
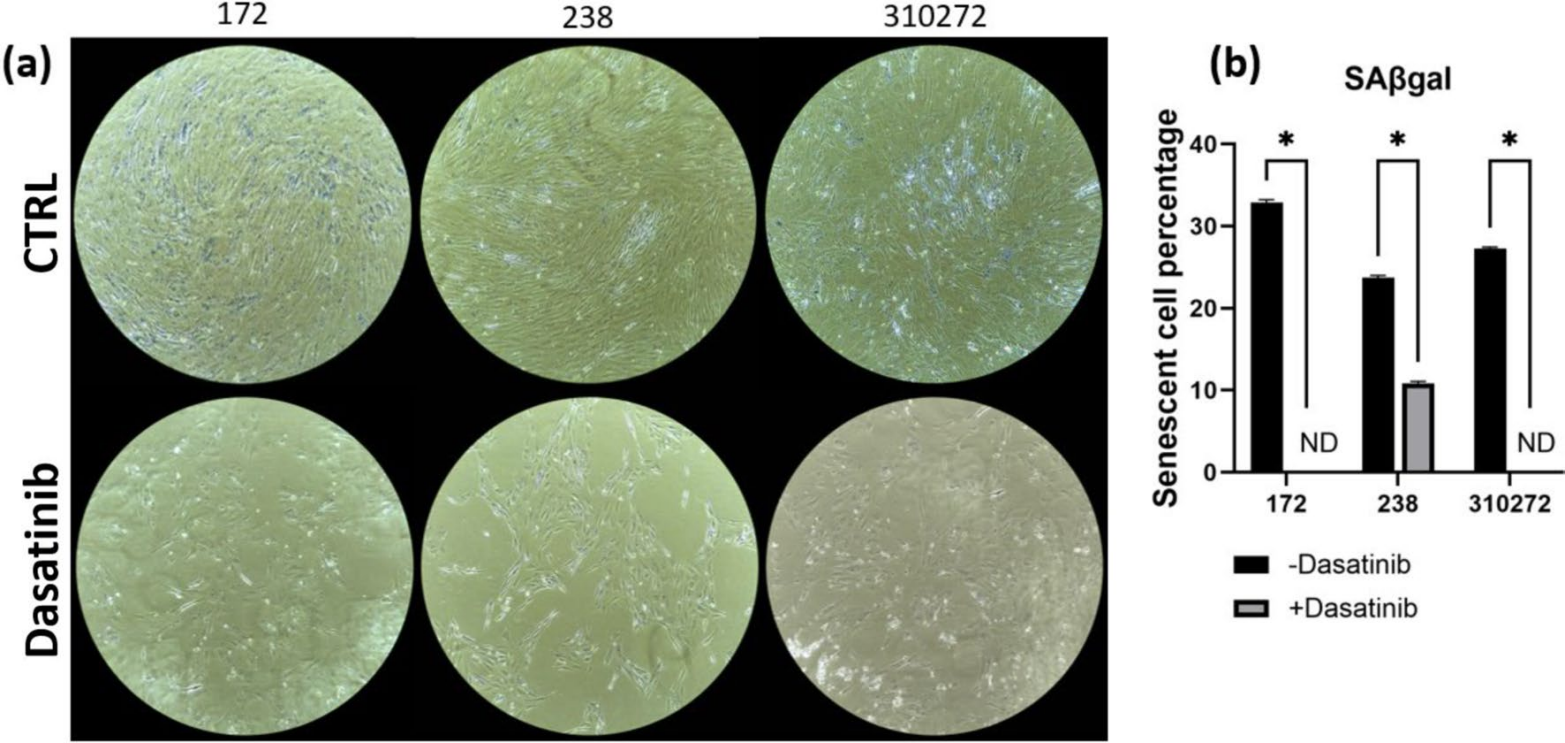
**a** 100 × magnification images of senescence staining with and without dasatinib treatment. (b) Percentage of senescent cells from stain images with and without treatment. Significance (two-tailed *t*-test): *P < 0.05. None detected (ND)

To show the growth of cells post-treatment, growth rate was assessed over 6 days following treatment of cells with/without dasatinib. Simple linear regression of the growth rate analysis (Fig. 1b) shows a positive trend in cells 6 days post-treatment, indicating increased growth. Senescent cell staining (Fig. 2) at the concentration of 2 µM shows a significant decrease in cell number with 0–10% of the remaining cells showing signs of senescence. From these results, we were able to establish a usable concentration of dasatinib for the other experiments of 2 µM as it clears a significant number of cells without being cytotoxic to the rest of the culture.

### Dasatinib Modulates Osteogenic Differentiation Capacities of MSCs

**Fig. 3:**
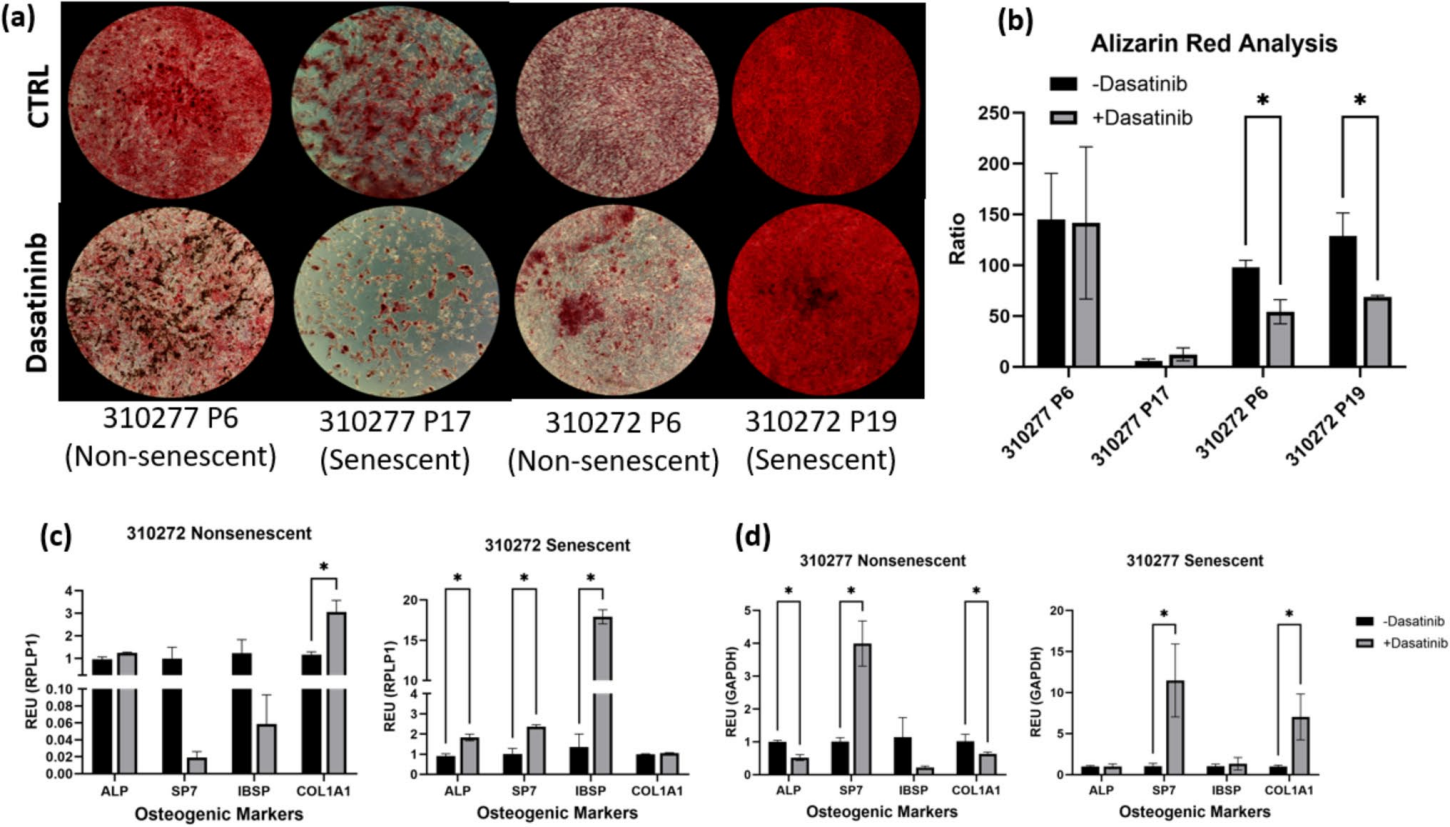
**a** Alizarin Red staining of osteogenic cultures after 21 days of differentiation. Images at 4X magnification. **b** Alizarin red concentration to cellular DNA concentration ratio. Significance (two-tailed *t*-test): *P < 0.05. **c** RT-PCR analysis of osteogenic markers on dasatinib treated MSCs from donor 310272. **d** RT-PCR analysis of osteogenic markers on dasatinib treated MSCs from donor 310277. Significance (*t*-test with multiple comparisons): *P < 0.05

Osteogenic differentiation of MSCs was performed to investigate how cells treated with dasatinib, both senescent and nonsenescent, reacted to the drug compared to cells not exposed to dasatinib. Osteogenic differentiation was phenotypically present for both the control and dasatinib treated wells (Fig. 3a). Further analysis of the stain concentration versus DNA content in each well (Fig. 3b) shows a consistent ratio of stain to DNA for 310277 while there is a significant decrease in the ratio for 310272. 310277 P6 and 310272 P6 showed significantly more mineralization visually when exposed to dasatinib compared to the non-treated control, which exhibited stain homogeneously through the entire well. 310277 P17 exhibited a significant decrease in cell number after exposure to dasatinib as senescent cells were eliminated, and the remaining cells exposed to dasatinib still show evidence of differentiation. To further investigate how dasatinib affects osteogenic differentiation, genetic expression was tested to research the markers for bone cells.

Real-time qPCR (Fig. 3c,d) showed osteogenic gene expression for both donors was significantly different between control and dasatinib-treated cells. Nonsenescent passages show a mixture of gene expression changes between genes. Notably, expression for COL1A1 for 310272 and SP7 for 310277 increased significantly, while expression for ALP and COL1A1 for 301277 decreased. However, in senescent passages, any significant change in gene expression increases after exposure to dasatinib. These results indicate that dasatinib appears to significantly increase osteogenic gene expression for senescent cultures of MSCs.

### Dasatinib Modulates Adipogenic Differentiation Capacities of MSCs

**Fig. 4:**
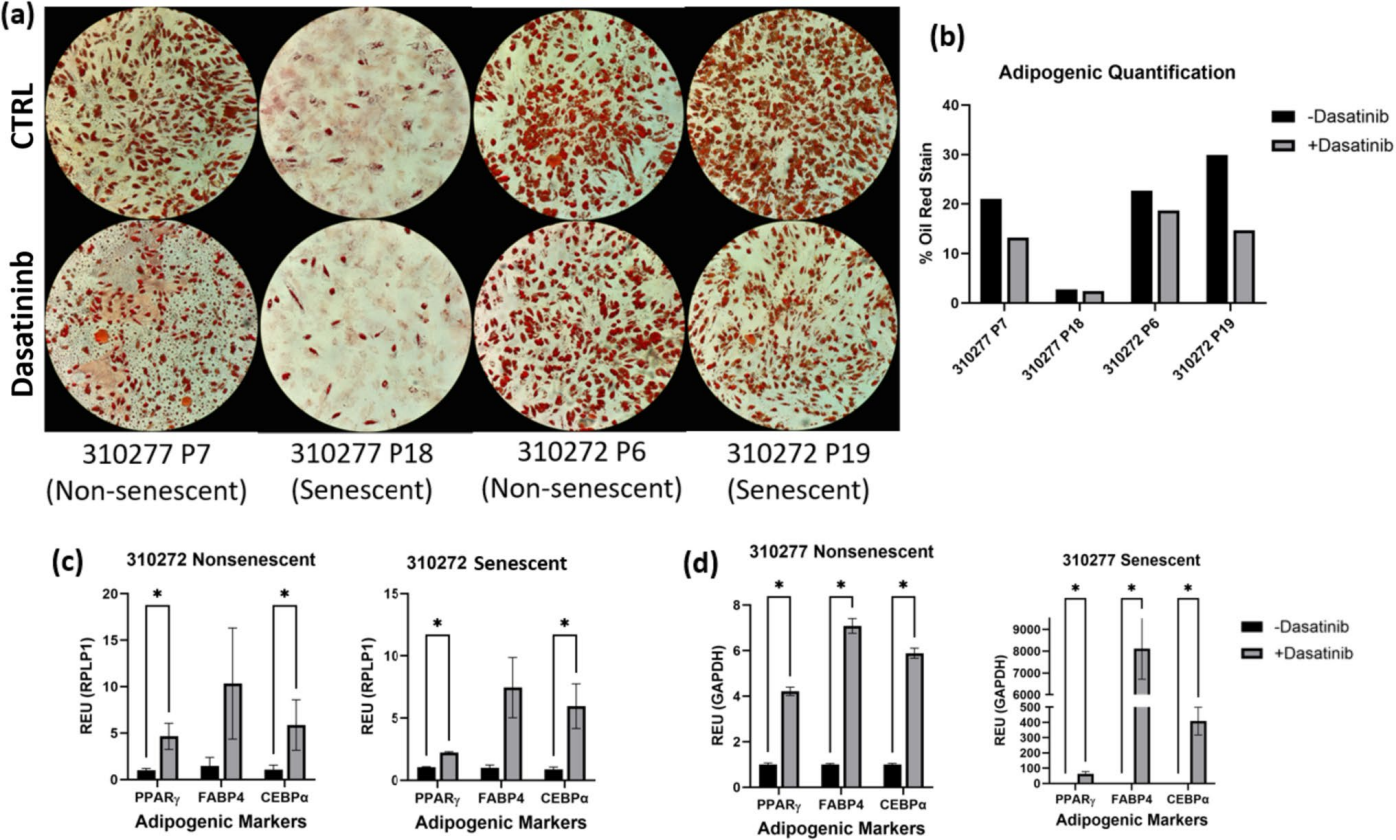
**a** Oil Red O staining of adipogenic cultures after 28 days of differentiation. Images at 10X magnification. **b** Quantification of adipogenic differentiation images based on percentage of stain per image. **c** RT-PCR analysis of adipose-specific genes on dasatinib treated MSCs from donor 310272. **d** RT-PCR analysis of adipose-specific genes on dasatinib treated MSCs from donor 310277. Significance (*t*-test with multiple comparisons): *P < 0.05

Adipogenic differentiation of MSCs was performed to investigate how cells treated with dasatinib, both senescent and nonsenescent, reacted to the drug compared to cells not exposed to dasatinib. Adipogenic differentiation showed no significant difference between the dasatinib treatment or DMSO normalized groups shown on (Fig. 4a). Lipid droplet formation and cell number on 310277 P7, 310272 P6, and 310272 P19 were nearly identical between both dasatinib and control groups. 310277 P18 exhibited little to no lipid droplet formation on both the control and dasatinib treated wells. To further investigate how dasatinib affects adipogenic cell differentiation, gene expression was tested to research the markers for adipose cells.

Real-time qPCR analysis showed dasatinib treatment significantly changed the adipogenic capacity of both donors. 310272 showed significant increases in PPARγ and CEBPα after exposure to dasatinib in both senescent and nonsenescent passages. Gene expression in 310277 showed significant increases in all markers tested after dasatinib exposure. These results indicate that dasatinib significantly increases adipogenic gene expression for all passages of MSCs (Fig. 4c,d).

**Fig. 5:**
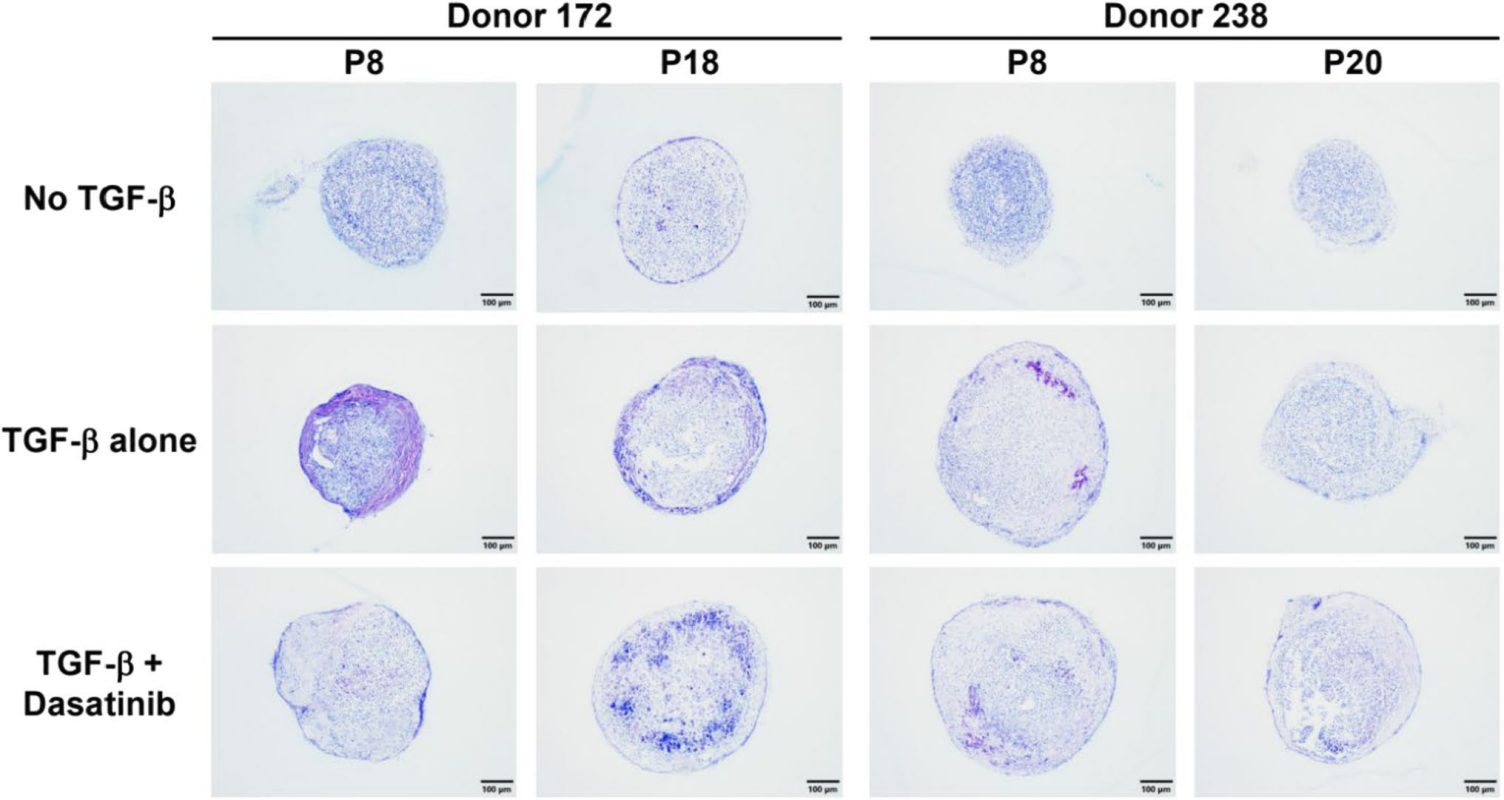
Toluidine blue stained images of MSC chondrogenic pellets after 28 days of differentiation. Images at 10 × magnification

### Dasatinib has No Clear Impact on the Chondrogenic Differentiation Capacity of MSCs

Chondrogenesis was performed to investigate how dasatinib affects the differentiation capacity of MSCs into chondrocytes. Toluidine blue staining (Fig. 5) produces a metachromatic shift from blue to purple reflecting binding to sulfated glycosaminoglycans (GAG) within the pellet’s extracellular matrix (ECM). This shift is most apparent on the rim of the pellets from 172 P8 TGF-β group. From our results, we can observe that dasatinib does not appear to have a consistent effect on either pellet size or GAG deposition; however, it is difficult to make a definitive conclusion due to low baseline levels of proteoglycan.

### Dasatinib’s Effect on IDO Activity

**Fig. 6:**
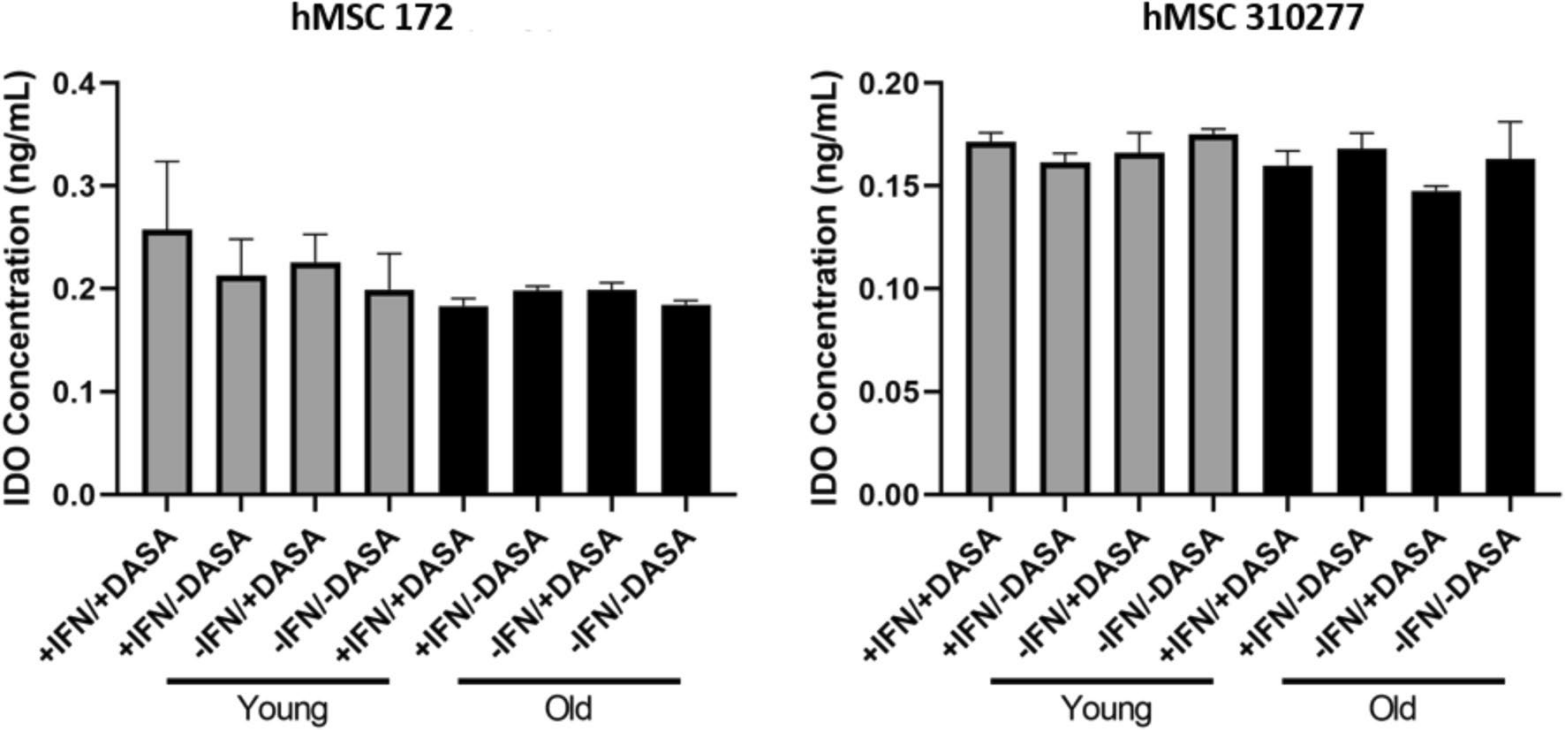
Combined IDO secretion from MSCs at early and late passage numbers, treated short-term with or without dasatinib and IFN-γ. Significance (One-way ANOVA): *P < 0.05

IDO assays were performed on MSCs exposed to dasatinib to investigate how dasatinib changes the immune response of MSCs. Cells were assessed for secretion of IDO upon short-term treatment with IFNγ and dasatinib. Figure 6 shows the combined data of both the supernatant and cell lysate. We note that there were no significant differences in the levels of IDO secretion for any of the experimental groups between senescent and nonsenescent cultures in both donors assessed.

## Discussion

Previous studies have tested the senolytic ability of other drugs [19] or assessed dasatinib alone for the senescence-associated secretory phenotype [20] and genetic makers associated with senescence [21]. This study is the first in-depth study to test the senolytic effects as well as post-treatment functionality of dasatinib alone on human mesenchymal stem cells in vitro. Dasatinib is known to block proliferation, adhesion, and migration of senescent cells *in vitro* via inhibition of tyrosine kinase [22, 23]. SABgal staining also showed that the cells being cleared out are predominantly senescent cells. While dasatinib has been effectively used to clear out senescent cells, the remaining live cells still had to be assessed for potency.

Established criteria for potency of MSCs state that cells must have the ability to differentiate into osteogenic, adipogenic, or chondrogenic cell lines [6, 24]. Osteogenesis stain and subsequent analysis against DNA concentration show that bone formation *in vitro* is not negatively affected by dasatinib. Further gene analysis shows that dasatinib has no effect or even increases the expression of certain bone-specific markers compared to the control samples for senescent passages. These results indicate that higher cell passages exposed to dasatinib have potentially higher potency than lower passages. Our osteogenic results agree with recent studies into regeneration of mouse bones using MSCs exposed to senolytics [25–27],  even when moving from using mouse derived MSCs to human MSCs.

Adipogenic staining results show similar conclusions that after dasatinib treatment, cells formed obvious lipid droplets that the oil red stain targeted. Further analysis of adipogenic genetic markers, after exposure to dasatinib, increased significantly across all markers for both senescent and nonsenescent cells. These results may indicate that dasatinib treatment should have a significantly higher stain concentration compared to the control, however, adipogenic staining took place much later than gene analysis. We can speculate that because of the time difference between these 2 data points, differential expression is minimized over time. It is worth noting that adipogenic potency does not diminish with progressive aging like osteogenic potency [28], regardless the increase in marker expression when exposed to dasatinib still indicates a significant increase in potency when compared to the control group. Adipogenic results from our study agree with another recent study investigating senolytic effects on chronic inflammation and metabolic function [23]. Our results are oriented towards *in vitro* human MSCs while this study measured age-related body mass changes when exposed to senolytics using mice as subjects.

Chondrogenic differentiation showed inconclusive results for MSCs treated with dasatinib. The TGF-β group appears to have higher GAG deposition in the pellet’s ECM, but pellets in the group treated with both dasatinib and TGF-β did not show a clear effect on pellet size or proteoglycan content. It is worth noting that reduced response of MSCs to the standard chondrogenic differentiation cocktail is consistent with previous literature, whereas the inclusion of a BMP stimulus along with TGF-β could have improved GAG deposition [29].

Immunomodulatory factors are another aspect of MSCs to investigate to define their potency [6]. Typical *in vitro* modulatory functions of MSCs are inhibition of T cell [30] and B cell proliferation. In addition, factors including IL10, indoleamine 2,3-dioxygenase (IDO), VEGF, CCL-5 or RANTES, prostaglandin E2, and nitric oxide (NO) are secreted by MSCs (either constitutively or by interaction with target cells) [24]. Since our IDO results show no difference in IDO secretion between young, old, treated, and untreated cells, there is no evidence showing compromised immunosuppressive ability of the MSCs. Therefore, dasatinib treated cells meet another requirement for MSC potency.

While we have provided a more expansive view of dasatinib and how it functionally improves ageing MSC cultures, more studies into how dasatinib affects telomere length *in vitro* (as telomeres tend to shorten when cells age causing loss of cell viability [31, 32]) would be beneficial to understanding how dasatinib affects aging. Investigation into mitochondrial dysfunction due to senescence could also provide a more full picture of dasatinib, as this dysfunction is another hallmark of the senescent phenotype [33, 34]. Another aspect of MSCs dasatinib could impact would be extracellular vesicle (EV) secretion. MSCs possess the ability to secrete EVs which are promising agents to treat various clinical conditions [35]. EV secretion is known to be upregulated in senescent cells [36] and dasatinib could impact this secretion and the factors associated with increased EV concentrations. These observations warrant further research on cellular phenotypes and other characteristics that are modulated with senolytic treatments.

## Conclusion

Dasatinib has significant effects on MSC function, notably on higher passages of cells. Dasatinib effectively eliminates senescent cells from higher passages. Osteogenic and adipogenic capacity is sustained or even increased with exposure to dasatinib shown in phenotypic and gene expression studies. Dasatinib treatment helps maintain the immunomodulatory capacity of MSCs as shown by IDO expression. Dasatinib could be an effective way to extend the lifespan of donor derived MSCs during *in vitro* MSC expansion and in clinical studies by treating higher passages with dasatinib mixed into maintenance media. Dasatinib can also be attributed to increasing the effectiveness of the cells for use in regenerative or immunomodulatory therapies. More research is suggested for dasatinib to investigate other aspects of immunosuppression such as inhibition of T cell and B cell proliferation as well as investigating the other criteria that define stem cell potency such as telomere length analysis.
