# Correction: Assessing the Effects of Dasatinib on Mesenchymal Stem/Stromal Cells

**DOI:** 10.1007/s12195-024-00833-y

**Published:** 2024-11-22

**Authors:** David P. Heinrichs, Vitali V. Maldonado, I. Kade K. Ardana, Ryan M. Porter, Rebekah M. Samsonraj

**Affiliations:** 1 Department of Biomedical Engineering, Engineering Research Center, College of Engineering, University of Arkansas, 700 W Research Center Boulevard, Fayetteville, AR 72701 USA; 2 Cell and Molecular Biology Interdisciplinary Program, University of Arkansas, Fayetteville, AR 72701 USA; 3 Department of Orthopedic Surgery, University of Arkansas for Medical Sciences, Little Rock, AR 72205 USA

**Correction to: Cellular and Molecular Bioengineering** 10.1007/s12195-024-00830-1

In the Acknowledgements, the National Institute of Health COBRE Funding Grant Number was missing. The Grant Number is P20GM125503.

